# Assessing delivery practices of mothers over time and over space in Uganda, 2003–2012

**DOI:** 10.1186/s12982-016-0049-8

**Published:** 2016-06-14

**Authors:** Daniel A. Sprague, Caroline Jeffery, Nadine Crossland, Thomas House, Gareth O. Roberts, William Vargas, Joseph Ouma, Stephen K. Lwanga, Joseph J. Valadez

**Affiliations:** Centre for Complexity Science, University of Warwick, Coventry, CV4 7AL UK; METRe Group, Department of International Health, Liverpool School of Tropical Medicine, Pembroke Place, Liverpool, L3 5QA UK; School of Mathematics, University of Manchester, Manchester, M13 9PL UK; Statistics Department, University of Warwick, Coventry, CV4 7AL UK; Management Sciences for Health, Kampala, Uganda; Management Sciences for Health, USAID STAR-E project, Kampala, Uganda

**Keywords:** Survey, Facility-based delivery, LQAS, Uganda, Spatial modelling, Ease of access, Early warning system

## Abstract

**Background:**

It is well known that safe delivery in a health facility reduces the risks of maternal and infant mortality resulting from perinatal complications. What is less understood are the factors associated with safe delivery practices. We investigate factors influencing health facility delivery practices while adjusting for multiple other factors simultaneously, spatial heterogeneity, and trends over time.

**Methods:**

We fitted a logistic regression model to Lot Quality Assurance Sampling (LQAS) data from Uganda in a framework that considered individual-level covariates, geographical features, and variations over five time points. We accounted for all two-covariate interactions and all three-covariate interactions for which two of the covariates already had a significant interaction, were able to quantify uncertainty in outputs using computationally intensive cluster bootstrap methods, and displayed outputs using a geographical information system. Finally, we investigated what information could be predicted about districts at future time-points, before the next LQAS survey is carried out. To do this, we applied the model to project a confidence interval for the district level coverage of health facility delivery at future time points, by using the lower and upper end values of known demographics to construct a confidence range for the prediction and define priority groups.

**Results:**

We show that ease of access, maternal age and education are strongly associated with delivery in a health facility; after accounting for this, there remains a significant trend towards greater uptake over time. We use this model together with known demographics to formulate a nascent *early warning system* that identifies candidate districts expected to have low prevalence of facility-based delivery in the immediate future.

**Conclusions:**

Our results support the hypothesis that increased development, particularly related to education and access to health facilities, will act to increase facility-based deliveries, a factor associated with reducing perinatal associated mortality. We provide a statistical method for using inexpensive and routinely collected monitoring and evaluation data to answer complex epidemiology and public health questions in a resource-poor setting. We produced a model based on this data that explained the spatial distribution of facility-based delivery in Uganda. Finally, we used this model to make a prediction about the future priority of districts that was validated by monitoring and evaluation data collected in the next year.

**Electronic supplementary material:**

The online version of this article (doi:10.1186/s12982-016-0049-8) contains supplementary material, which is available to authorized users.

## Background

### Maternal mortality, neonatal deaths and stillbirths

In 2010, an estimated 287,000 women died as a result of pregnancy or delivery-related complications [1]. Over 99 % of these maternal deaths occurred in developing countries [2]. Ranking 161 of the 187 UN member nations in the 2013 Human Development Index, Uganda is one of the least developed countries in the world [3, 4]. At 360 maternal deaths per 100,000 live births in 2013, the maternal mortality ratio (MMR) is one of the highest in the world, more than 22 times higher than more developed regions where the MMR is estimated at 16 deaths per 100,000 live births [5].

Between 1995 and 2009, 2.7 million third-trimester stillbirths occurred yearly [1]. The majority of these deaths took place in developing countries and most are preventable with appropriate antenatal, delivery and postnatal care [6]. In Uganda, the neonatal mortality rate was 26 per 1000 live births in 2010, while the estimated stillbirth rate was 25 per 1000 live births. About half of stillborns are classified as intrapartum deaths–deaths which occur during labour or delivery. A woman in a low-income country in sub-Saharan Africa is 24 times more likely to suffer an intrapartum stillbirth than a woman from a high income country [1].

### Skilled birth attendance and place of delivery

Skilled birth attendance (SBA) is associated with reduced maternal and neonatal mortality and risk of stillbirth [1, 7–9]. In low-income countries, newborns delivered by a skilled birth attendant in a health facility stand a greater chance of survival than newborns delivered elsewhere [7, 10]. Effective SBA requires an environment enabling skilled attendants to perform to the best of their abilities and gives them access to essential medications and equipment and timely intervention or referral options in the event of complications [11].

Promoting skilled birth attendance in health facilities is a global priority, especially to achieve the targets set for Sustainable Development Goal (SDG) 3— Ensure healthy lives and promote well-being for all at all ages—and to meet targets of other global initiatives, such as the Every Woman, Every Child initiative [12, 13]. The Ugandan government’s priority strategies to combat maternal and child mortality include improving access to emergency obstetrical care and developing an enabling environment for SBA [14, 15]. Facility-based delivery (FBD) is a proxy for lower-risk delivery as delivery with SBA can be difficult to quantify when patients assume that all clinicians are skilled.

### Factors associated with facility-based deliveries

Studies exploring factors associated with using health facilities for delivery have included: maternal characteristics, index pregnancy characteristics, access, socio-cultural beliefs and past experiences of the mother. Maternal characteristics such as young maternal age, high levels of education and increased autonomy are positively associated with FBD [16–20]. A cross-sectional study in the district of Busia identified that parity less than four [AOR 2.9 (1.6–5.6)] and autonomy in deciding to attend ANC [AOR 1.9 (1.1–3.4)] are positively associated with FBD [18]. A large proportion of women less than 20 years old (65.8 %), with secondary education or above (81.4 %), in the highest wealth quintile (87.7 %), and mothers of first-order births (73.1 %) report delivering in an institutional setting [20].

Several traits relating directly to the index pregnancy also affect safe delivery practices. The timing of the onset of labour and duration of labour do impact delivery location. Labour onset late at night or short duration of labour can inhibit a mother from accessing a health facility for delivery services [21]. A cross-sectional study conducted in Bugesera district in Rwanda reports that attending more ANC visits (OR 1.567 [1.163–2.112]) is positively associated with FBD [22], while a study in southern Tanzania highlights that having been advised on FBD during ANC [AOR 1.82 (1.25–2.63)] is positively associated with skilled attendance at delivery [23].

Ease of physical and financial access to health facilities is positively associated with FBD [18, 19, 22, 24, 25]. Increased distance to facilities or facilities located in difficult terrain, as well as high costs, both formal and informal, mitigate against FBD [16, 17, 23, 26–30]. A study conducted in four districts of Laos in 2009 reveals that eliminating user fees associated with delivery at the point of services increases by 9.8 percentage point (p < 0.1) the coverage of skilled birth attendance [26]. A cross-sectional study from the 2011 Nepalese DHS showed that increase distance to facilities or facilities located in difficult terrain explained up to 1.7 %-point of the 17.7 %-point regional gap in FBD between terai/hill and mountainous regions [28]. The 2008–2009 Indian district level household and facility survey revealed that the mean out-of-pocket expenditure on delivery care increased by 7 % for every 10 % increase in state domestic product per capita [29]. High levels of absenteeism and lack of supervision of health care workers are known barriers to using health facilities [31].

A woman’s knowledge of pregnancy and delivery-related risks also support using a health facility [1, 9, 11, 18, 20, 32]. A mother’s previous birth experiences, location of the penultimate birth, her beliefs regarding health providers’ skills and her perceptions of the quality of health facilities are additional factors influencing her decision to use FBD [16–19, 22, 24, 27, 31, 33]. A woman’s supposition of how she will be received by health care workers and if her wishes will be respected by them also influence uptake of FBD [18, 20, 32, 34, 35]. Traditional beliefs, including the fatalistic perception that maternal death is a normal risk and to be expected, lead to underutilisation of health facilities [1, 15, 34, 35].

### Monitoring maternal and neonatal health indicators

Uganda, a Millennium Countdown Country, is one of 75 countries where >95 % of maternal and child deaths between 1990 and 2010 took place; these mortality rates are high nationwide [1]. The World Health Organisation and UNICEF recommend that countries analyse data at both national and subnational levels to identify gaps and inequities in health services. Both UN agencies also urge improvements in surveillance and survey data to decrease missing data [1].

It is also important to uncover factors influencing FBD. The studies mentioned above generally include at most one geographic covariate, and most do not consider the spatial distribution of FBD. The aim of our study is to identify the simultaneous correlates of FBD, in order to provide a framework for prioritizing districts for support. Our study assesses variations over time and space in FBD and fits a statistical model to identify factors associated with FBD. We apply this model to 2003–2011 data to identify areas expected to have low indicator coverage in 2012 and validate this prediction with the 2012 data. This approach can therefore inform policy-makers and program managers on the status of FBD and trends and variations occurring over time and can identify locations needing further investigation.

## Methods

### Data collection and sampling

The study was conducted by the USAID STAR E-LQAS project, which is implemented  Management Sciences for Health with Liverpool School of Public Health as a technical partner for LQAS. Trained district health managers collected data from individuals with household surveys conducted in 19–64 districts of Uganda at seven points in time during 2003–2012, using the Lot Quality Assurance Sampling (LQAS) methodology [35]. The surveys were financed by the World Bank and USAID [36] with questions adapted from accepted sources such as the Uganda Demographic Surveys. The District Health Management Team divided each district into 4–6 administrative subdistrict strata called supervision areas (SA) and selected 19 mothers of children 0–11 months (or 24 if 4 SAs) randomly from each SA. The SA sample size was selected so that when subdistrict data (the SA) are aggregated, the resulting district-level coverage proportion estimates for key indicators are calculated with a 95 % confidence interval not exceeding ±10 %. Villages were selected using probability proportional to size (PPS) sampling, wherein a comprehensive village population list supplied by each district was the sampling frame used to select villages from which the individual samples are taken. There was on average 88 villages in the sampling frame of each SA. PPS sampling ensures that sample villages are selected based upon their proportional representation of the entire population. Usually a sample of 19 villages was identified, sometimes less if some villages had a large population size relative to others in the same SA. Individual respondents were then randomly selected from the PPS-selected villages using a randomizing technique [35]. The main approached used was segmentation sampling. Segmentation was recommended as it was found to be a more rigorous second-stage sampling technique [37] and is now advocated in several survey guidelines [38–40]. District Health Officers also requested a second approach be offered, namely, simple random sampling from an updated village listing of households. The latter was recommended only in cases in which a recently updated list existed and could be verified. With either approach once a reference house was selected the next closes house was selected for interview. This addition reduced the chance of a house having a zero probability of selection. The former approach was recommended in the trainings and used most frequently. Table 1 shows the number of districts in each Ugandan region that were surveyed in each year and the number of mothers interviewed in those regions. A total of 18,471 randomly selected mothers of children aged 0–11 months were interviewed, the inclusion criterion being that mothers had have been present in the village at least 3-months prior to the survey. Each maternal questionnaire included demographic characteristics and various health-related behaviours. Respondents with missing or erroneous responses were removed, leaving a total of 18,098 (98 %) records with complete information. These data were integrated into a superset, and in this study we analysed mothers’ responses to the question “Where did you give birth?”, their age at the time of the survey (in years) and their education level (none, primary, secondary, post-secondary). Uganda LQAS data reliability studies are available for review [41, 42].

**Table 1:** Number of districts and mothers surveyed within each region of Uganda for each survey year

Survey	Region	Total no. districts	No. districts surveyed	No. mothers surveyed
2003	Central	13	6	627
	Eastern	15	5	493
	Northern	13	2	303
	Western	15	6	681
	Total	56	19	2104
2004	Central	13	1	95
	Eastern	15	4	380
	Northern	13	4	380
	Western	15	2	190
	Total	56	11	1045
2006	Central	13	4	380
	Eastern	15	3	284
	Northern	13	2	245
	Western	15	3	286
	Total	56	12	1195
2009	Eastern	24	4	419
	Total	80	4	419
2010	Eastern	32	9	969
	Western	26	14	1427
	Total	112	23	2396
2011	Central	24	8	798
	Eastern	32	16	1712
	Northern	30	2	190
	Western	26	18	1864
	Total	112	44	4564
2012	Central	24	13	1368
	Eastern	32	21	2282
	Northern	30	7	684
	Western	26	23	2414
	Total	112	64	6748
Total				18,471

We obtained district-level data from a variety of sources, including geospatial road and population data from 2009 [34] and 2010 Geographical Information System (GIS) locations of health centres. We calculated the number of health facilities per capita (per 100,000 inhabitants) based on the number of health facilities with in-patient beds (level III and above), since mothers are referred to these higher-level facilities for FBD. Household assets data from DHS 2011 [20] were used to stratify responses by economic quintiles. Altitude data was obtained from the US Geological Survey [43].

### Data analysis

Our analysis consists of 3 phases: FBD mapping, model construction, and prediction of priority districts and population strata in them. Phases 1–2 used the 2003–2011 data, while phase 3 also included the 2012 data. All analysis was done using the statistical software R version 2.15 [44]; we used the R-package ‘maptools’ [45] to construct the maps.

#### FBD mapping

We classified mothers as giving birth either at home or in a health facility and plotted on a map the percentage of mothers with FBD for each district surveyed. One map was produced for each cluster of survey years: 2003–2004, 2006, 2009–2010, and 2011. Survey years were combined so that a similar number of surveyed districts were included in each map. We calculated 95 % confidence intervals (CI) using clustered bootstrapping [46], a non-parametric error estimation method which takes into account residual spatial correlation of the indicator (See Appendix 1 for a detailed description of how the maps and confidence intervals were constructed). We use a clustered bootstrap because it accounts for the fact that the survey samples were clustered within supervision areas. The total population size of each supervision area was not available so this analysis gives an equal weighting for each supervision area.

#### Model construction

Using all 2003–2011 data, we fitted a logistic regression model to investigate factors simultaneously associated with FBD. The individual-level factors included in the model were age, education and the year that the mother was surveyed. We also included district-level covariates: each mother was assigned a value for the number of health facilities per capita, population density, road density, wealth index, and mean and standard deviation of the altitude of her district. Mothers were also assigned a categorical variable specifying whether or not they lived in Kampala, to correct for the fact that Kampala had extremely different district-level covariates to all other districts and should therefore be considered separately. Covariates with significant nonlinearity were base-2-log-transformed before being incorporated into the model (see “Appendix 2” for the reasoning). All covariates were included as continuous variables, except for education, which was categorical. We used forward selection based [47] on the Akaike Information Criterion (AIC) to include interaction terms between the covariates if they improved the model. This is one of the standard procedure for model selection.

Tables 2 and 3 display information about each covariate: the distribution of ages and educational categories for the mothers, and the average values and range of the district-level covariates calculated over all 112 districts in Uganda.

**Table 2:** Characteristics of individual-level covariates (sample sizes)

Variable	Levels	2003–2004	2006	2009–2010	2011	2012
Education	None	623	180	1542	2506	3924
	Primary	1929	727	729	1110	1645
	Secondary	507	239	319	644	848
	Post-secondary	63	33	83	180	267
	Total	3122	1179	2673	4440	6684
Age	<20	724	265	530	843	825
	20–30	1765	645	1507	2522	3820
	30–40	580	251	567	965	1785
	>40	53	18	69	110	254
	Total	3122	1179	2673	4440	6684

**Table 3:** Characteristics of district-level covariates, over all the districts surveyed

Variable	Min	25 % Q	Median	75 % Q	Max
Wealth index	−2.0	−0.6	−0.1	0.4	3.6
Health centres per capita (per 100,000 inhabitants)	0.2	1.8	3.7	5.8	36.7
Road density (metres per km^2)^	0	87	125	170	359
Standard deviation of altitude (m)	10	31	64	162	956
Mean altitude (m)	701	1071	1143	1307	2428
Population density (per km^2^)	4	64	122	241	8647

As a first stage to validate our selected model, we compared it to a null spatial model, for which the probability of FBD for a mother is predicted to be the average value for her district. This null model represents a situation where the differences between the indicators in each district are not captured by any covariates and are assumed to be random. The model with the lowest AIC is the better construct.

As a second stage of model validation, we constructed a Receiver Operational Characteristic (ROC) curve. The ROC curve plots the relationship between the true positive rate (the probability that a true outcome is correctly predicted to be true) and the false positive rate (the probability that a false outcome is predicted to be true) for different classification cutoffs. The accuracy can be summarised by the area under the ROC curve (AUC). An AUC of 1.0 indicates a perfect prediction: all datapoints were correctly classified. An AUC of 0.5 indicates a random test, which allocates positive outcomes at random half of the time [48].

As a third stage, we used two-fold Monte Carlo cross-validation [49] to estimate the prediction error for unseen data; the model was repeatedly fitted to a randomly chosen half of the 2003–2011 data and then used to predict the FBD values of the other half. For each iteration, we calculated the squared error between the observed and predicted district-level FBD indicator, and took the mean over all 1000 iterations. The square-root of the resulting mean squared error defines a prediction error for each district with the same units as the original indicator, and thus is a standard estimate of the absolute difference between the prediction and the indicator.

Our selected model gave an estimate of the odds ratio (OR) for FBD for each covariate. For our model, the OR for a covariate is the ratio between the odds of FBD for two mothers, both of whom, for the covariate being examined, have all other covariates set to their average values. If the covariate is categorical, such as education level, then the ratio is between each level and the lowest level, which, in this example, is ‘no formal education’. If a base-2-log-transformed covariate was used in the model, then the ratio is between the odds calculated for the covariate and double the covariate. For the other continuous covariates, the ratio is between the odds calculated for the covariate and the covariate plus a unit increase. The OR therefore provides an estimate for how strongly each covariate is associated with the odds of FBD.

#### Prediction of priority groups

Finally, we used the model to classify unsurveyed districts into ‘priority’ groups to flag districts predicted to have particularly low indicator values. Since we do not know the distribution of age and education in these unsurveyed districts, we decided to predict an upper and lower limit of a range of values for the indicator in each district rather than an average value. We chose the values for age and education most strongly associated with FBD and then the values with the strongest negative association, and then we used the model to predict the probability of FBD for a mother with her age and education set to these values and the survey year set to 2012.

To obtain an estimate for the upper limit for the indicator in each district, we applied the model to the most strongly associated age and education values. To account for any uncertainty in the model parameters we took the upper part of 95 % CI obtained from the model with bootstrap clustering as a conservative estimate of the upper limit. For the lower limit, the same procedure was carried out with the negatively associated values and taking the lower part of the 95 % CI. The lower and upper limit define the predicted range for each district. The priority groups were assigned on the basis of these limits.

The low-priority group, defined as districts with lower limits between 50–100 % FBD and upper limits between 80–100 % FBD, contained districts that were likely to have high indicator values. The mild-priority group, defined as districts with lower limits between 0–30 % and upper limits between 60–80 %, contained districts likely to have fairly low indicator values. The high-priority group, defined as districts with lower limits between 0–30 % and upper limits between 30–60 %, contained districts likely to have very low indicator values. All other scenarios were classified as an unclear-priority group. We then validated the projections by checking that the 2012 values lay within their predicted ranges.

## Results

### Facility-based deliveries 2003–2011

**Fig. 1:**
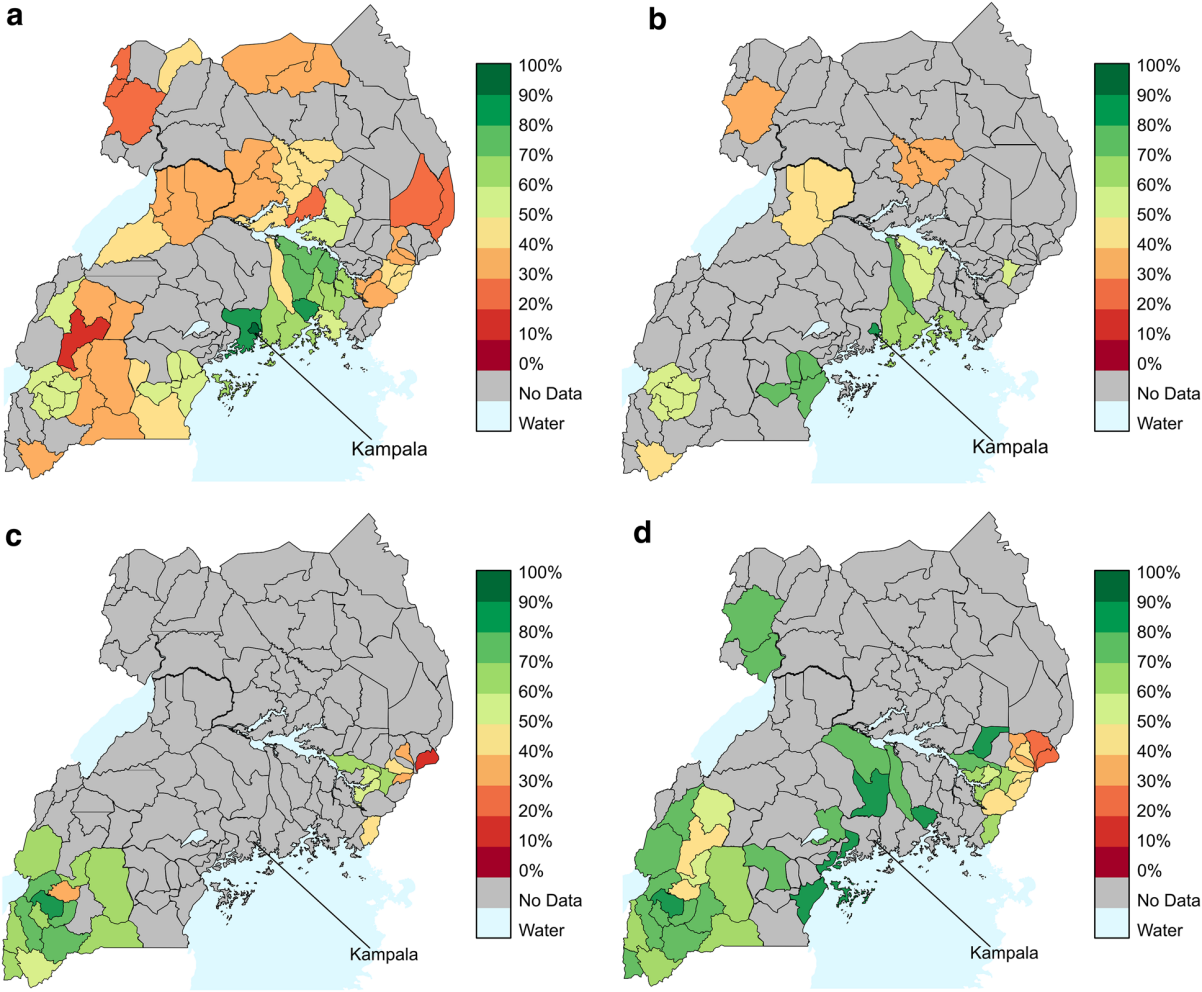
Maps of the indicator. Percentage of mothers of children aged 0–11 months that gave birth in a health facility for **a** 2003–2004, **b** 2006, **c** 2009–2010, **d** 2011. 95 % confidence intervals for the indicator are ±14.3  % or lower. Data for 2003 and 2004, and for 2009 and 2010 have been combined for these maps due to the small number of districts surveyed in 2004 and 2010. In the rest of the analysis they are separated

We plotted spatially the percentage of mothers of children aged 0–11 months with FBD for different points in time (Fig. 1) and report the confidence intervals in Additional file 1: Table S1 (Appendix 1). During the time period over which the data were collected, some districts subdivided; to aid comparisons across time we plotted the indicator on the 2011 district map. The mean of the indicator across all districts in each year was 49 % in 2003–2004, 57 % in 2006, 58 % in 2009–2010, 65 % in 2011, and 66 % in 2012. For early survey years (2003–2006) most districts had <60 % of mothers of infants with FBD (77 and 58 % of the districts surveyed in 2003–2004 and 2006 respectively), except around the capital, Kampala (95 % in 2003–04 and 89 % in 2006). For later years (2009–2011), >60 % of mothers had FBD in most surveyed districts (61 and 68 % of the districts surveyed in 2009–2010 and 2011 respectively). Although much of northern Uganda was not sampled in the more recent time periods, the one district sampled in both 2003–2004 and 2011, Arua, increased from 26.3 to 73.4 % (Additional file 1: Table S1). The progress has not been replicated as dramatically everywhere, with half of the eastern Ugandan districts surveyed in 2011, for example, still reporting <50 % FBD.

### Logistic regression model

All district-level covariates except for wealth index and mean altitude showed significant nonlinearity, and so were log-base-2 transformed before being incorporated in the model. Marital status was removed from the model since it did not have a statistically significant effect. Age was included as a continuous variable because the AIC value for this model was lower than models with 2, 3, and 5 age categories and the same as the model with 4 age categories.

Table 4 shows the logistic regression results. The second column reports the estimated effect of each covariate or interaction of covariates on FBD on the logistic scale. The third column reports the odd ratio of FBD related to each covariate. Each odds ratio was calculated between two ‘average’ mothers: one who has a secondary education, is 25-years old, was surveyed in 2007, and lives in a district where all district-level covariates take their average values (as given in Table 3); and the other who is identical except for a unit increase in the covariate in question. The results show that the odds of FBD were significantly lower for each one-year increase in maternal age [OR 0.98, 95 %CI (0.97, 0.99)], whereas they were significantly increased for each additional level of maternal education [primary: OR 1.59 (1.42, 1.78), secondary: OR 3.37 (2.88, 3.94), post-secondary: OR 10.4 (6.28, 18.1)]. The odds of FBD were significantly greater in districts with double the health facilities per capita [OR 1.12 (1.02, 1.23)], or road density [OR 1.13 (1, 1.26)], or in districts with a unit increase in the wealth index [OR 1.38 (1.24, 1.53)]. Living in the capital, Kampala, was strongly associated with FBD [OR 8.38 (2.24, 23)]. Districts with a double unit increase in the standard deviation of altitude (a proxy for the roughness and difficulty of the terrain) were strongly associated with a decrease in the odds of FBD [OR 0.89 (0.84, 0.94)]. Finally, there was a significant time trend: mothers surveyed in later years were more likely to have FBDs [OR 1.08 (1.04, 1.13)].

**Table 4:** Logistic regression model for delivery in a health facility in Uganda

Covariates	Coefficient and 95 % CIs	Odds ratio and 95 % CIs
(Intercept)	0.107 [− 0.303, 0.503]	–
Age	−0.0285 [− 0.0406, −0.0162]^a^	0.98 [0.974, 0.987] ^a^
Education (primary)	0.408 [0.215, 0.607]^a^	1.59 [1.42, 1.78] ^a^
Education (secondary)	1.42 [1.14, 1.7]^a^	3.37 [2.88, 3.94] ^a^
Education (post)	2.72 [1.98, 3.94]^a^	10.4 [6.28, 18.1] ^a^
Health facilities per capita^b^	−0.036 [− 0.314, 0.254]	1.12 [1.02, 1.23] ^a^
Road density^b^	0.0824 [0.002, 0.156]^a^	1.13 [1, 1.26] ^a^
Population density^b^	0.297 [0.0743, 0.525]^a^	0.97 [0.892, 1.06]
Living in Kampala	1.9 [0.808, 3.14]^a^	8.38 [2.24, 23] ^a^
District wealth index	0.307 [0.208, 0.415]^a^	1.38 [1.24, 1.53] ^a^
Standard deviation of altitude^b^	−0.176 [− 0.26, −0.0921]^a^	0.89 [0.842, 0.941] ^a^
Mean altitude	0.325 [0.072, 0.566]^a^	1 [0.9997, 1.001]
Year of survey	0.0777 [0.0164, 0.142]^a^	1.08 [1.04, 1.13] ^a^
*Interaction terms*		
Standard deviation of altitude: mean altitude	−0.191 [−0.28, −0.0998]^a^	–
Health facilities per capita: year	0.0538 [0.0134, 0.0956]^a^	–
Road density: population density	−0.253 [−0.372, −0.145]^a^	–
Mean altitude: year	−0.0696 [−0.105, −0.0329]^a^	–
Population density: year	−0.0854 [−0.119, −0.0506]^a^	–
Health facilities per capita: mean altitude	−0.0962 [−0.333, 0.122]	–
Education (primary): year	0.0133 [−0.0183, 0.0441]	–
Education (secondary): year	−0.052 [−0.0977, −0.00668]^a^	–
Education (post): year	−0.119 [−0.281, −0.00558]^a^	–
Age: year	0.00213 [0.000132, 0.0041]^a^	–
Health facilities per capita: mean altitude: year	0.0446 [0.0149, 0.0765]^a^	–

The second column gives the coefficient for each term included in the model. The third column gives the odds ratio between two ‘average’ mothers with unit difference in the covariate, both mothers aged 25 and with secondary-level education, surveyed in 2007, and all district-level covariates set to their average

^a^ *A 95* %-*significant positive or negative effect.* Confidence intervals were calculated using clustered bootstrapping with 1000 iterations

^b^Results for a doubling of this variable, rather than a unit increase

Our model had significantly lower AIC (AIC = 13,383) than the null spatial model (AIC = 13,690), and hence was a better model of the observed spatial variation. The area under the ROC curve reported a 71 % probability that our model ranked a true positive data point higher than a true negative one, which is significantly better than the 50 % probability predicted by a random model. Using cross-validation, the indicator predicted for each district based on unseen data was within 20 percentage points of the observed indicator for 95 % of the districts, and 77 % of the districts were within 15 percentage points of the observed indicator.

### Predicting facility-based delivery in unsurveyed districts

**Fig. 2:**
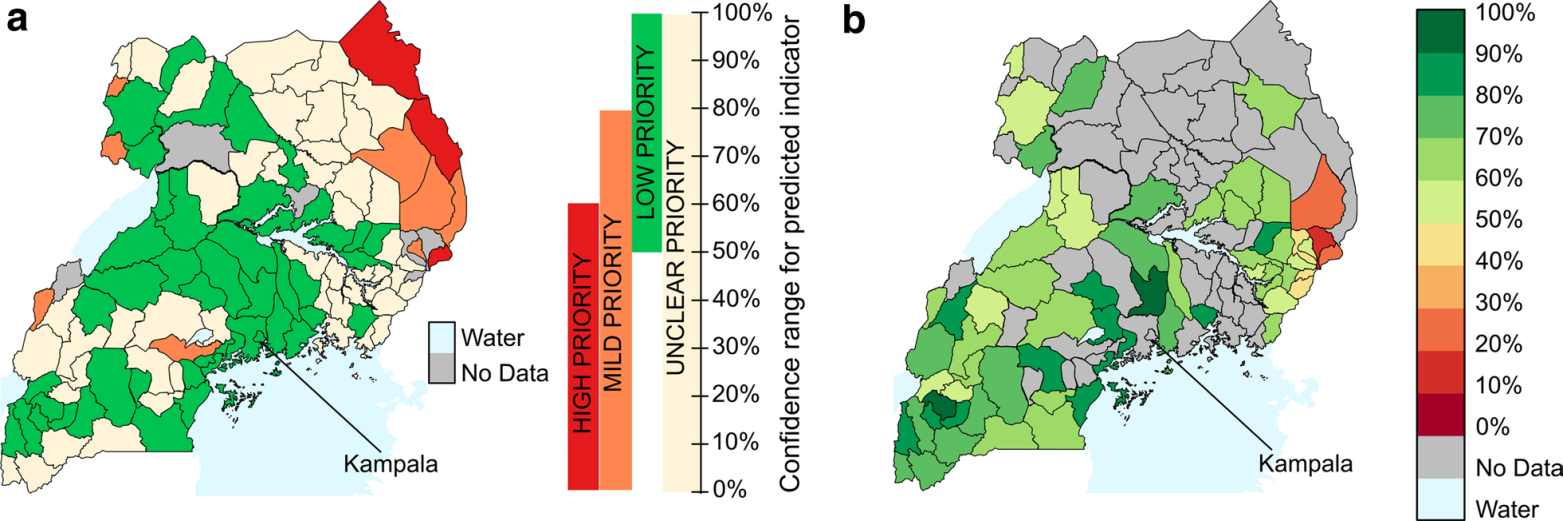
Predicted priority map and comparison with unseen data. **a** Priority Map for districts in 2012. Districts are assigned high, mild, low, or unclear priority based on the confidence interval predicted by the model. Kampala, for example, has a predicted indicator confidence interval between 50 and 100 %, and is therefore assigned a low priority. **b** Indicator for 2012 as observed in a subsequent LQAS survey. The model was not fitted using this data, and so this map provides an independent test of the predicted confidence interval. All surveyed districts in 2012 have indicator values within the predicted confidence interval, and districts in Eastern Uganda that were predicted to be mild or high priority are observed to have very low values for the indicator

Using the 2003–2011 fitted logistic regression model, we predicted for all Ugandan districts the reasonable lower and upper limits for FBD during 2012. We used 18 (50) years old as most strongly (negatively) associated age value, and post-secondary (none) as most strongly (negatively) associated education value. We categorised districts into different priority levels using these predicted ranges. We validated these predicted priority groups by comparing them with indicator values calculated from a subsequent 2012 LQAS survey covering 61 districts (Fig. 2). Additional file 1: Table S2 in Appendix 2 gives the details of the predicted range and the observed indicator in 2012. The priority map identifies many north-eastern districts as being mild or high priority—classifications that agree with the low values for these districts seen in the observed data. In addition, all but four (6.6 %) of the observed indicator values fall within the predicted range.

## Discussion

Our results show the percentage of mothers with FBD increasing over time, but varying among surveyed districts (Fig. 1). This variation is strongly correlated with geographic and demographic factors. Initiatives meant to increase the uptake of services, including maternal services, have been introduced in Uganda in the past several years [50]. Although this analysis cannot prove causation, the trend over time displays a progressively greater uptake of maternity services in most areas. Despite this overall time trend, the survey data show that some districts display low FBD, particularly in north-eastern Uganda. The logistic regression model provides possible explanations; specifically, low uptake is associated with low health facility density, low road density, mountainous terrain, and lack of geographical access to health facilities due to few roads. In other countries, these same factors have been shown to have the same impact on uptake of maternal services [17, 27, 28, 51–54]. Furthermore, previous research in Uganda shows that “difficult-to-access” areas also suffer acute staffing shortages, high rates of absenteeism, and poor quality of care [15, 55], potentially reducing demand by women in labour.

We also found that age, level of education and district wealth status also influence FBD. Younger women and those with higher levels of education are more likely to practice FBD. Our findings are consistent with the 2011 Uganda DHS, which reports that older mothers are less likely to give birth in a health facility [20]. However, this result is inconsistent with a meta-analysis of socio-geographic factors in numerous countries, which found age to have no statistical significance in determining FBD; it did find, however, that high parity is negatively associated with FBD [56, 57]. Studies controlling for parity have found either no effect of maternal age on FBD or that increased age was positively related to increased use of delivery services [53]. We were unable to control for parity in our study. As parity is often linked to maternal age, it may be the influence of parity, rather than age, which we have vicariously detected. In our study, increased maternal education was positively associated with FBD, a finding consistent with other studies [20, 51, 52, 56–59]. We also determined that mothers in wealthier districts were more likely to use FBD. This finding is consistent with the results of numerous other studies reporting wealth and economic access to health care as facilitators behind FBD [16, 17, 26, 27, 29, 30, 56].

We validated the logistic regression model in three ways: the AIC for the model was significantly lower than that of the null spatial model, the area under the ROC curve for the model was 71 % (which can be considered fair predictive power), and cross-validation showed that for 95 % of districts the model prediction was within 20 % of the ‘true’ indicator value. Given the context of this last prediction, and comparing these values with the largest uncertainty in the observed indicator, 11 %, we think that this model shows fair predictive power.

Although the national average increased from 49 % in 2003–2004 to 66 % in 2012, the wide range of subnational results indicated gaps in equitable access to health services. In 2011, for example, the Eastern region district prevalence ranged from 23 % in Bukwa district to 82 % in Kumi district. Although these two districts contribute to regional and national prevalence, the district prevalence is indicative of the inequitable occurrence of FBD in the districts. A study in Ghana found similar variations, further highlighting the importance of detecting subregional variation when planning health programs and allocating resources to decrease the gaps [60]. To achieve equitable access to services, subregional variation must be detected and addressed [1, 61].

Our study also examined an additional practical use of logistic regression, namely, assigning predicted priorities to districts based on the lowest expected value for FBD. By using the model to construct the predicted range of FBD in each district, we can identify districts, prior to a survey, most likely to need intervention. This method can be used to suggest which areas should be included in the next LQAS survey. By excluding districts which are very likely to have high indicator values (the ‘low priority’ districts), policy-makers could concentrate surveys in districts which have uncertain priority or which are very likely to have low indicator values. Such an approach could help lessen the gaps and inequities in maternal health care and help Uganda identify health system changes needed to decrease both maternal and child mortality [1]. This feature of logistic regression suggests it can be used as a kind of early warning system to detect priority districts in need of special attention.

### Limitations

The factors assessed in this study are not necessarily an exhaustive list of factors impacting uptake of FBD. We had limited data regarding household and personal wealth, for example, and despite free healthcare in Uganda, FBD has costs which reduce access to care [25, 62]. Additional data on maternal wealth could increase our understanding of the relationship between cost and use of FBD. District-level data was limited to one time point for each covariate, so the model relies on the assumption that the district-level covariates did not change significantly over time. This assumption is likely to be more reasonable for some covariates than others: mean and standard deviation of altitude will not have changed over the course of these surveys, but population density is more likely to have changed. Without supervision area locations it was not possible to calculate each supervision area’s distance to the nearest health facility, which is likely to be an important factor in FBD. We used health facilities per capita as the best proxy available for this variable.

We did not have data for parity in the assessment of maternal age and its impact on FBD. Further research should take into consideration their relationship and extricate their individual effects.

A hierarchical model may provide an alternative approach to estimate district-level information, as it could uncover a hidden ‘ease of access’ variable influencing FBD. Other studies attempting this approach [63] have tended to use data with greater spatial resolution than was available in this study. The model presented in this paper was designed to be fitted and interpreted by the survey collection teams as a regular part of monitoring and evaluation; hierarchical models would have introduced additional complexity to both fitting and interpretation. While these types of models may be investigated in the future, we showed by cross-validation that the current model provides a good fit without using hierarchical modelling.

The use of the model to predict the indicator in unsurveyed districts before an LQAS survey has been performed is of course limited by the lack of individual-level covariates in those districts. Since both age and education are strong predictors of FBD, it was not possible to give a point estimate for the indicator. However this method can be used to suggest which districts it would be most efficient to include in the next round of LQAS surveys.

## Conclusion

In this study we described and modelled the spatial and temporal patterns of an important health system indicator: percentage of mothers of children aged 0–11 months with FBD. Using data collected with LQAS across Uganda during 2003–2011, we plotted the time and regional variations of this indicator. The prevalence of FBD generally increases with time but remains low in some areas. The logistic regression model provides evidence that the likelihood of FBD is greater in districts with more health facilities per capita, more road infrastructure and higher wealth index; however, the use of FBD is lower among older women and greater for mothers with higher levels of education.

 The observational nature of this study cannot demonstrate causation but suggests that mothers *do* deliver in health facilities if the facilities are available and accessible. In addition, the model has the potential to provide a predicted range for the indicator in unsurveyed districts, and therefore can *flag* priority districts, which are likely to have low indicator values and require new surveys to assess the accuracy of this accuracy of the flag.

Finally, to understand maternal health related behaviour in Uganda and specifically to appreciate the factors involved in seeking FBD, the findings of this research should be assessed together with studies of the capacities of health facilities to provide EmOC, and with qualitative research analysing belief systems and experience which influence care seeking.

## Additional file


10.1186/s12982-016-0049-8 Supplementary material.
