# Supplementary material for: Assessing delivery practices of mothers over time and over space in Uganda, 2003–2012
Source: Emerg Themes Epidemiol. 2016 Jun 14;13:9. doi: 10.1186/s12982-016-0049-8 (PMC4908697; doi:10.1186/s12982-016-0049-8)
Supplement: Supplementary file 1 — 10.1186/s12982-016-0049-8 Supplementary material. [file 12982_2016_49_MOESM1_ESM.docx]

## Appendix 1: Construction of the maps

We used the R package ‘maptools’ [38] to construct the maps by colouring the districts in a map of Uganda based on the percentage of mothers surveyed in each district who had given birth in a health facility. One map was produced for each cluster of survey years: 2003-04, 2006, 2009-10, and 2011. Survey years were combined so that a similar number of districts were surveyed in each cluster: only 11 districts were surveyed in 2004 and 4 in 2009.

One complication for producing these maps was that the number of districts in Uganda increased between 2004 and 2006, and between 2006 and 2009. The indicator for each district was calculated based on the boundaries used at the time of the survey, but to aid comparison between survey years we plotted the indicator for all survey years on the post-2009 district map. For the older districts that were divided in subsequent years, we applied the value of the indicator for the old district to each of the districts in 2009 that it had been divided into. For example, the 2006 district in the Western region of Uganda called ‘Bushenyi’ was subsequently divided into five districts. When plotting a map of the 2006 survey results, we assigned the indicator value of the 2006 ‘Bushenyi’ district to all of the five districts that it became in the 2009 map.

We used a clustered bootstrap to calculate confidence intervals, because a simple bootstrap assumes that data originate from independent samples. Here, the data are clustered by supervision area and are therefore not independent. Supervision area codes were not provided in the pre-2009 data, so responses in these years were assigned to a cluster based on their subcounty.

The procedure for performing the clustered bootstrap is as follows:

1. Original data consists of g clusters,
2. Repeat:
   1. Create a new data set by sampling g clusters with replacement from G
   2. Estimate the parameter of interest, such as the mean, based on this new sample.
3. Calculate the [2.5, 97.5]^th^ percentiles of the parameter. This corresponds to the 95% confidence interval for the estimate of population parameter.

**Table S1:** Indicator and confidence interval for all surveyed districts.

| Survey Year | District | Indicator | Lower 95% CI | Upper 95% CI |
| --- | --- | --- | --- | --- |
| 2003-04 | APAC | 35.8% | 30.8% | 42.7% |
|  | ARUA | 26.3% | 21.3% | 33.1% |
|  | BUSHENYI | 58.8% | 54.7% | 68.5% |
|  | HOIMA | 42.1% | 35.0% | 51.0% |
|  | IGANGA | 65.3% | 56.3% | 73.3% |
|  | JINJA | 84.2% | 79.7% | 87.9% |
|  | KABALE | 38.9% | 34.7% | 42.1% |
|  | KABAROLE | 57.9% | 52.6% | 68.2% |
|  | KABERAMAIDO | 27.4% | 22.6% | 33.4% |
|  | KALANGALA | 69.5% | 59.3% | 73.7% |
|  | KAMPALA | 94.7% | 94.1% | 98.2% |
|  | KAMULI | 70.2% | 65.3% | 78.1% |
|  | KAMWENGE | 16.0% | 13.0% | 21.0% |
|  | KAYUNGA | 49.5% | 40.2% | 51.3% |
|  | KITGUM | 32.6% | 26.6% | 36.1% |
|  | KYENJOJO | 34.5% | 28.4% | 40.8% |
|  | LIRA | 40.9% | 32.9% | 47.4% |
|  | MASAKA | 59.6% | 56.3% | 67.5% |
|  | MASINDI | 38.9% | 30.2% | 45.3% |
|  | MAYUGE | 50.0% | 37.4% | 60.9% |
|  | MBALE | 43.2% | 40.0% | 51.4% |
|  | MBARARA | 39.1% | 33.5% | 43.0% |
|  | MOYO | 44.2% | 39.4% | 47.9% |
|  | MUKONO | 69.3% | 63.4% | 75.9% |
|  | NAKAPIRIPIRIT | 21.1% | 15.7% | 27.1% |
|  | RAKAI | 43.6% | 35.5% | 44.0% |
|  | SIRONKO | 35.8% | 27.3% | 45.7% |
|  | SOROTI | 54.7% | 45.6% | 61.9% |
|  | TORORO | 35.8% | 32.9% | 42.9% |
|  | WAKISO | 89.4% | 82.7% | 92.2% |
| 2006 | ARUA | 35.6% | 31.8% | 41.7% |
|  | BUSHENYI | 54.7% | 49.1% | 59.2% |
|  | KABALE | 44.2% | 36.3% | 54.5% |
|  | KAMPALA | 89.4% | 86.4% | 94.3% |
|  | KAMULI | 53.7% | 45.5% | 60.6% |
|  | KAYUNGA | 71.3% | 67.5% | 76.3% |
|  | LIRA | 34.5% | 28.5% | 43.4% |
|  | MASAKA | 70.2% | 60.5% | 77.1% |
|  | MASINDI | 47.4% | 42.3% | 53.3% |
|  | MAYUGE | 67.0% | 61.1% | 75.6% |
|  | MBALE | 52.1% | 49.2% | 57.6% |
|  | MUKONO | 67.4% | 60.5% | 73.0% |
| 2009-10 | BUDAKA | 56.5% | 48.6% | 69.4% |
|  | BUDUDA | 34.6% | 29.2% | 40.2% |
|  | BUHWEJU | 36.5% | 31.5% | 42.5% |
|  | BUKWA | 16.7% | 13.0% | 23.5% |
|  | BUSHENYI | 80.0% | 74.3% | 87.9% |
|  | BUSIA | 49.7% | 41.4% | 52.9% |
|  | BUTALEJA | 55.2% | 51.3% | 60.6% |
|  | IBANDA | 69.0% | 62.7% | 75.7% |
|  | ISINGIRO | 61.1% | 53.6% | 69.8% |
|  | KABALE | 51.9% | 41.4% | 58.1% |
|  | KANUNGU | 68.4% | 62.3% | 76.7% |
|  | KAPCHORWA | 32.3% | 22.8% | 40.8% |
|  | KASESE | 61.1% | 56.0% | 67.4% |
|  | KIRUHURA | 61.1% | 55.2% | 66.9% |
|  | KISORO | 62.3% | 54.6% | 68.5% |
|  | MBALE | 63.1% | 60.1% | 67.6% |
|  | MITOOMA | 65.3% | 62.1% | 72.2% |
|  | NTUNGAMO | 70.2% | 64.2% | 73.8% |
|  | PALLISA | 65.9% | 59.8% | 71.4% |
|  | RUBIRIZI | 76.0% | 72.6% | 84.9% |
|  | RUKUNGIRI | 70.5% | 62.6% | 82.2% |
|  | SHEEMA | 78.9% | 71.5% | 90.0% |
|  | SIRONKO | 47.6% | 42.8% | 60.1% |
| 2011 | ARUA | 73.4% | 67.4% | 79.7% |
|  | BUDAKA | 54.3% | 46.6% | 67.6% |
|  | BUDUDA | 45.1% | 41.7% | 56.5% |
|  | BUHWEJU | 44.8% | 30.5% | 56.8% |
|  | BUKWA | 22.9% | 16.3% | 31.1% |
|  | BULAMBULI | 33.7% | 27.3% | 42.9% |
|  | BUSHENYI | 85.3% | 78.8% | 90.4% |
|  | BUSIA | 61.1% | 54.8% | 72.7% |
|  | BUTALEJA | 63.4% | 60.5% | 71.7% |
|  | IBANDA | 59.3% | 54.3% | 64.3% |
|  | ISINGIRO | 60.0% | 49.9% | 65.3% |
|  | JINJA | 86.4% | 83.7% | 88.8% |
|  | KABALE | 61.4% | 55.5% | 72.0% |
|  | KABAROLE | 72.8% | 62.1% | 81.5% |
|  | KALANGALA | 81.1% | 72.8% | 87.9% |
|  | KAMWENGE | 48.9% | 41.0% | 53.4% |
|  | KANUNGU | 68.4% | 62.1% | 74.0% |
|  | KAPCHORWA | 48.4% | 44.1% | 50.8% |
|  | KASESE | 71.6% | 68.9% | 79.5% |
|  | KAYUNGA | 71.3% | 70.2% | 76.8% |
|  | KIBUKU | 61.7% | 53.1% | 69.3% |
|  | KIRUHURA | 67.4% | 65.5% | 76.1% |
|  | KISORO | 71.9% | 66.4% | 75.4% |
|  | KUMI | 82.3% | 77.5% | 87.3% |
|  | KWEEN | 29.0% | 22.4% | 35.5% |
|  | KYENJOJO | 57.9% | 51.1% | 63.6% |
|  | LUWERO | 86.8% | 84.4% | 91.9% |
|  | MANAFWA | 47.4% | 40.5% | 55.1% |
|  | MASAKA | 80.5% | 75.9% | 87.4% |
|  | MBALE | 62.1% | 57.1% | 67.6% |
|  | MBARARA | 74.6% | 70.6% | 77.2% |
|  | MITOOMA | 74.7% | 69.4% | 83.2% |
|  | MITYANA | 79.8% | 72.8% | 85.0% |
|  | MPIGI | 86.2% | 78.8% | 89.9% |
|  | NAKASONGOLA | 72.3% | 66.4% | 80.8% |
|  | NEBBI | 75.8% | 73.1% | 82.7% |
|  | NTUNGAMO | 72.8% | 69.5% | 79.0% |
|  | PALLISA | 72.6% | 68.9% | 77.7% |
|  | RUBIRIZI | 70.8% | 60.8% | 79.3% |
|  | RUKUNGIRI | 76.8% | 69.1% | 80.3% |
|  | SHEEMA | 75.8% | 68.6% | 81.9% |
|  | SIRONKO | 47.4% | 42.1% | 58.8% |
|  | SSEMBABULE | 71.3% | 60.8% | 79.7% |
|  | TORORO | 49.1% | 43.0% | 55.1% |

## Appendix 2: Nonlinearity of the covariates

Some of the covariates included in the model showed significant nonlinearity. We reduced this by applying a base-2 logarithm transformation and checking if the AIC for the fitted model was reduced. Four covariates—health facilities per capita, population density, road density, and standard deviation of altitude—were transformed in this way (shown in Figure S1). Two covariates—mean altitude and wealth index—were not improved by the transformation and were thus left untransformed (Figure S2).

**Figure S1:** Comparison of raw and log-transformed covariates.

**Figure S2:** Covariates that were not transformed.

**Table S2:** Prediction interval and actual observed indicator for 2012. Indicators that were outside the prediction interval are marked in red.

| District | Lower Prediction | 2012 indicator | Upper prediction |
| --- | --- | --- | --- |
| ABIM | 44% | - | 85% |
| ADJUMANI | 43% | 73% | 88% |
| AGAGO | 47% | - | 87% |
| ALEBATONG | 34% | - | 78% |
| AMOLATAR | 56% | - | 90% |
| AMUDAT | 18% | - | 64% |
| AMURIA | 44% | 61% | 83% |
| AMURU | 63% | - | 94% |
| APAC | 59% | 72% | 91% |
| ARUA | 59% | 51% | 91% |
| BUDAKA | 41% | 62% | 82% |
| BUDUDA | - | 49% | - |
| BUGIRI | 58% | - | 89% |
| BUHWEJU | 29% | 59% | 75% |
| BUIKWE | 73% | - | 94% |
| BUKEDEA | 50% | 68% | 86% |
| BUKOMANSIMBI | 46% | - | 85% |
| BUKWA | 12% | 24% | 51% |
| BULAMBULI | - | 46% | - |
| BULIISA | 75% | 58% | 97% |
| BUNDIBUGYO | 26% | 63% | 78% |
| BUSHENYI | 71% | 91% | 95% |
| BUSIA | 48% | 64% | 85% |
| BUTALEJA | 47% | 65% | 84% |
| BUTAMBALA | 61% | - | 90% |
| BUVUMA | 42% | - | 97% |
| BUYENDE | 42% | - | 83% |
| DOKOLO | - | - | - |
| GOMBA | 29% | - | 77% |
| GULU | 58% | - | 90% |
| HOIMA | 58% | 66% | 91% |
| IBANDA | 48% | 65% | 85% |
| IGANGA | 50% | - | 86% |
| ISINGIRO | 48% | 65% | 85% |
| JINJA | 58% | 85% | 90% |
| KAABONG | 10% | - | 51% |
| KABALE | 41% | 71% | 82% |
| KABAROLE | 48% | 81% | 85% |
| KABERAMAIDO | 57% | 65% | 89% |
| KALANGALA | 71% | 80% | 94% |
| KALIRO | 49% | - | 85% |
| KALUNGU | 66% | - | 92% |
| KAMPALA | 57% | - | 97% |
| KAMULI | 61% | - | 91% |
| KAMWENGE | 40% | 61% | 81% |
| KANUNGU | 55% | 82% | 89% |
| KAPCHORWA | 18% | 53% | 61% |
| KASESE | 43% | 72% | 83% |
| KATAKWI | 40% | 66% | 83% |
| KAYUNGA | 55% | 68% | 88% |
| KIBAALE | 56% | 64% | 89% |
| KIBOGA | 67% | 86% | 93% |
| KIBUKU | 36% | 59% | 79% |
| KIRUHURA | 65% | 73% | 92% |
| KIRYANDONGO | 37% | - | 83% |
| KISORO | 46% | 72% | 84% |
| KITGUM | 50% | - | 88% |
| KOBOKO | 49% | 55% | 87% |
| KOLE | 37% | - | 80% |
| KOTIDO | 34% | 65% | 78% |
| KUMI | 51% | 85% | 86% |
| KWEEN | - | 12% | - |
| KYANKWANZI | 51% | - | 89% |
| KYEGEGWA | 35% | - | 80% |
| KYENJOJO | 50% | 57% | 86% |
| LAMWO | 41% | - | 91% |
| LIRA | 70% | - | 94% |
| LUUKA | 45% | - | 83% |
| LUWERO | 67% | 93% | 93% |
| LWENGO | 41% | 68% | 83% |
| LYANTONDE | 55% | - | 88% |
| MANAFWA | 31% | 44% | 75% |
| MARACHA | 16% | - | 62% |
| MASAKA | 76% | 81% | 96% |
| MASINDI | 83% | 59% | 98% |
| MAYUGE | 48% | - | 86% |
| MBALE | 48% | 68% | 86% |
| MBARARA | 74% | 80% | 95% |
| MITOOMA | 52% | 84% | 87% |
| MITYANA | 49% | 84% | 86% |
| MOROTO | 9% | - | 49% |
| MOYO | 65% | - | 93% |
| MPIGI | 76% | 83% | 96% |
| MUBENDE | 44% | 62% | 83% |
| MUKONO | 72% | 79% | 94% |
| NAKAPIRIPIRIT | 18% | 21% | 74% |
| NAKASEKE | 55% | - | 90% |
| NAKASONGOLA | 65% | 77% | 94% |
| NAMAYINGO | 38% | - | 86% |
| NAMUTUMBA | 50% | - | 86% |
| NAPAK | 17% | - | 69% |
| NEBBI | 68% | 71% | 94% |
| NGORA | 59% | - | 90% |
| NTOROKO | - | - | - |
| NTUNGAMO | 40% | 73% | 81% |
| NWOYA | - | - | - |
| OTUKE | 38% | - | 84% |
| OYAM | 44% | - | 84% |
| PADER | 49% | - | 88% |
| PALLISA | 59% | 67% | 90% |
| RAKAI | 60% | 66% | 90% |
| RUBIRIZI | 61% | 56% | 92% |
| RUKUNGIRI | 58% | 76% | 90% |
| SERERE | 53% | - | 87% |
| SHEEMA | 58% | 85% | 90% |
| SIRONKO | 34% | 57% | 79% |
| SOROTI | 41% | 69% | 82% |
| SSEMBABULE | 33% | 80% | 81% |
| TORORO | 37% | 53% | 79% |
| WAKISO | 63% | - | 93% |
| YUMBE | 44% | - | 87% |
| ZOMBO | 19% | - | 67% |
